# Drug interaction prediction using ontology-driven hypothetical assertion framework for pathway generation followed by numerical simulation

**DOI:** 10.1186/1471-2105-9-S6-S11

**Published:** 2008-05-28

**Authors:** Takeshi Arikuma, Sumi Yoshikawa, Ryuzo Azuma, Kentaro Watanabe, Kazumi Matsumura, Akihiko Konagaya

**Affiliations:** 1 Department of Computer Science, Tokyo Institute of Technology, 2-12-1 Oookayama, Meguro, Tokyo, Japan; 2 Genomic Sciences Center, RIKEN, 1-7-22 Suehiro, Tsurumi, Yokohama, Kanagawa, Japan

## Abstract

**Background:**

In accordance with the increasing amount of information concerning individual differences in drug response and molecular interaction, the role of *in silico *prediction of drug interaction on the pathway level is becoming more and more important. However, in view of the interferences for the identification of new drug interactions, most conventional information models of a biological pathway would have limitations. As a reflection of real world biological events triggered by a stimulus, it is important to facilitate the incorporation of known molecular events for inferring (unknown) possible pathways and hypothetic drug interactions. Here, we propose a new Ontology-Driven Hypothetic Assertion (OHA) framework including pathway generation, drug interaction detection, simulation model generation, numerical simulation, and hypothetic assertion. Potential drug interactions are detected from drug metabolic pathways dynamically generated by molecular events triggered after the administration of certain drugs. Numerical simulation enables to estimate the degree of side effects caused by the predicted drug interactions. New hypothetic assertions of the potential drug interactions and simulation are deduced from the Drug Interaction Ontology (DIO) written in Web Ontology Language (OWL).

**Results:**

The concept of the Ontology-Driven Hypothetic Assertion (OHA) framework was demonstrated with known interactions between irinotecan (CPT-11) and ketoconazole. Four drug interactions that involved cytochrome p450 (CYP3A4) and albumin as potential drug interaction proteins were automatically detected from Drug Interaction Ontology (DIO). The effect of the two interactions involving CYP3A4 were quantitatively evaluated with numerical simulation. The co-administration of ketoconazole may increase AUC and Cmax of SN-38(active metabolite of irinotecan) to 108% and 105%, respectively. We also estimates the potential effects of genetic variations: the AUC and Cmax of SN-38 may increase to 208% and 165% respectively with the genetic variation UGT1A1*28/*28 which reduces the expression of UGT1A1 down to 30%.

**Conclusion:**

These results demonstrate that the Ontology-Driven Hypothetic Assertion framework is a promising approach for *in silico *prediction of drug interactions. The following future researches for the *in silico *prediction of individual differences in the response to the drug and drug interactions after the administration of multiple drugs: expansion of the Drug Interaction Ontology for other drugs, and incorporation of virtual population model for genetic variation analysis, as well as refinement of the pathway generation rules, the drug interaction detection rules, and the numerical simulation models.

## Background

The role of *in silico *prediction of drug interactions on the pathway level is becoming more and more important for solving real-world problems. Multiple-drug regimens exemplify the need for the computer-assisted prediction of drug interactions. Multiple-drug regimens are commonly prescribed for elderly patients suffering from more than one disease. However, they sometimes cause unexpected severe side effects because of the drug interactions or individual differences concerning response to the drugs [1]. Therefore, the prediction of drug interactions for preventing the side effects is an important issue for these regimens.

On the other hand, information useful for *in silico *drug interaction prediction has increased very rapidly in recent years. Technological innovations in genomic sciences have produced an enormous amount of biomolecular information including sequences, structures, and pathways. In order to integrate the biomolecular information, ontology is attracting a lot of attention [2,3]. In addition, pharmacokinetics modeling and simulation are emerging, promising techniques to understand the dynamic behavior of drug metabolic pathways [4,5].

To develop a practical *in silico *drug interaction prediction system by integrating the above information and techniques, the following issues must be solved.

### Context dependency of drug-metabolic pathways

Drug-metabolic pathways do not exist a priori. They strongly depend on contexts and situations including the administration route, single nucleotide polymorphism (SNP) of drug-response genes, and the administration of multiple drugs and foods. Therefore, a dynamic reconstruction of drug metabolic pathways from primitive molecular events is necessary for drug interaction prediction on the pathway level. Such reconstruction requires the formal definition of molecular events and the relations among them, i.e., the Drug Interaction Ontology (DIO).

### Treatment of multiscale events

Pathways triggered by drug administration consist of multiscale events: from the molecular level to the body level, ranging from nanoseconds to hours or days in terms of drug response. For example, drug administration and drug excretion are body-level events, while drug transport and enzymatic reactions are molecular-level events. A comprehensive view from the molecular level to the body level is necessary in order to understand multi-scale events.

### Quantitative evaluation of interactions

Quantitative evaluation plays an essential role to estimate the degree of side effects caused by drug interactions. More than one drug interactions may occur in drug-metabolic pathways from the qualitative reasoning view point. However, not all drug interactions cause side effects because of differences in binding affinity and molecular population. Quantitative simulation models with an *in silico *drug interaction prediction system must be incorporated to discriminate serious drug interactions from negligible ones. It should be also noted that total drug metabolism depends on not only kinetic parameters but also physiological parameters such as organ volumes and blood flows. Incorporation of kinetic parameters and physiological parameters is necessary for a realistic simulation model to recapture experimental data.

In this paper, we propose a new drug-interaction prediction framework called "Ontology-Driven Hypothetic Assertion (OHA)" focusing mainly on automatic generation of drug-metabolic pathways, automatic detection of drug interactions on multiple-drug regimens [6], and quantitative evaluation of the drug interactions with numerical simulation. The effectiveness of the OHA framework was demonstrated in the prediction of interactions between irinotecan (CPT-11) and ketoconazole.

### Design philosophy

#### Ontology-Driven Hypothetical Assertion

The Ontology-Driven Hypothetical Assertion framework maps inferences onto the Drug Interaction Ontology as both machine and human understandable form. The inferences include the results of qualitative reasoning and numerical simulation, i.e. generated pathways and detected drug interactions and generated differential equations and simulation results. The assertions enable to interpret the obtained results such as drug interaction candidates, simulation models, the pharmacokinetic moment values with background knowledge on pharmaceutical science and biochemistry in the ontology.

#### Causality-based pathway modularization

Modularization is necessary for the dynamic reconstruction of pathways that depend on dose conditions. Careful selection of primitive modules is the key to ensuring the soundness of pathway reconstruction. Molecular events, such as molecular transport and enzymatic reactions, are well-formed primitive modules for this purpose. In this paper, we refer to the primitive modules as "molecular events", and the aggregation of molecular events as "pathways".

To avoid redundant pathway branch constructions, which are non-essential for the target drug interactions, we adopt causality-based modularization in which each molecular event is defined by the unique relationship between key molecules before and after the event. The triadic relationship <trigger, situator, resultant>, proposed by Yoshikawa et al. [7], is one such causality that can be commonly found in molecular reactions. For example, in case of enzymatic reactions, substrates, enzymes and other products correspond to trigger, situator and resultant, respectively. In the case of molecular transport, extra (intra) cellular molecules, transporters and intra (extra) cellular molecules correspond to the participants of the triadic relation, respectively. The triadic relationship can be applicable to higher level events like drug dosage and drug excretion, as long as its causality is unique and clear.

**Figure 1:**
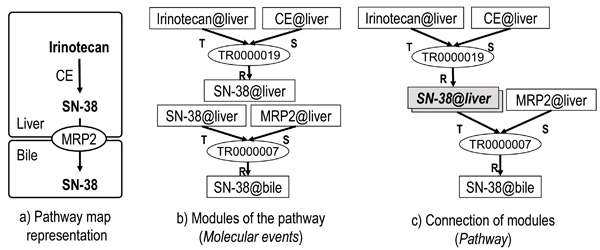
**Decomposition of a pathway with primitive molecular events**. a) Ordinary pathway map representation. b) Primitive molecular events including trigger (T), situator (S) and resultant (R). c) Aggregation of molecular events at SN-38@liver. Rectangles and ellipses are instances of continuants and processes, respectively, in b) and c).

Figure 1 (a)(b)(c) shows a simple example of pathway reconstruction with two primitive molecular events: an enzymatic reaction in which carboxylesterase (CE) metabolizes irinotecan into SN-38 (7-ethyl-10-hydroxycamptothecin) in the liver, and molecular transport in which SN-38 in the liver is transported to the bile by MRP2 (Multidrug resistance-associated protein 2). Two molecular events are connected at the resultant of the enzymatic reaction (TR0000019) and the trigger of the molecular transport (TR0000007) for passing SN-38 in the liver (SN-38@liver) to the bile.

#### Ontology-based knowledge base construction

The ontological approach in knowledge base design is adopted for resource sharing and the semantic description of molecular events and pathways. Ontology is necessary to define molecular events and pathways in a form that can be shared among computers and human beings. This enables the full use of powerful computational intelligence for dynamic pathway reconstruction in a way that human intelligence can follow and understand. Ontology is also important for establishing interoperability among web resources and thereby to make use of the latest drug reaction information published in the semantic web [2,8]. Public biological ontologies, especially in the field of chemical biology, are now dramatically increasing, and have a great potential to develop sustainable knowledge bases for molecular reaction and pathways.

**Figure 2:**
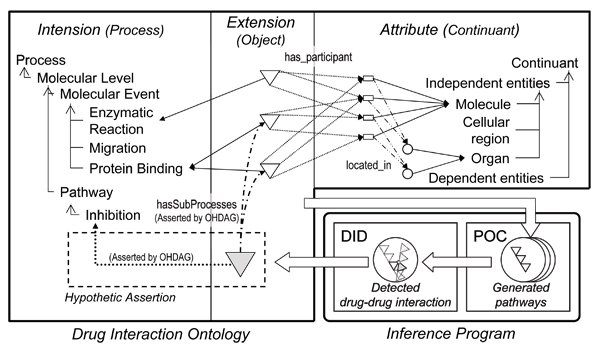
**Overview of the Ontology-Driven Hypothetic Assertion (OHA) framework**. Drug Interaction Ontology (DIO) provides triple views for events and pathways, namely, intension, extension, and attribute. White triangles in extension are molecular events such as CYP3A4 mediated metabolism of irinotecan. White rectangles and circles in attribute are molecules and organs respectively; the organs indicate the location of molecules. The gray triangles and arrowed dashed lines represent the hypothetic assertions generated by the OHA framework. The Pathway Object Constructor (POC) generates pathways by connecting molecular events. The Drug Interaction Detector (DID) detects drug interactions from the generated pathways and asserts the interactions as hypothetic assertions into DIO.

**Figure 3:**
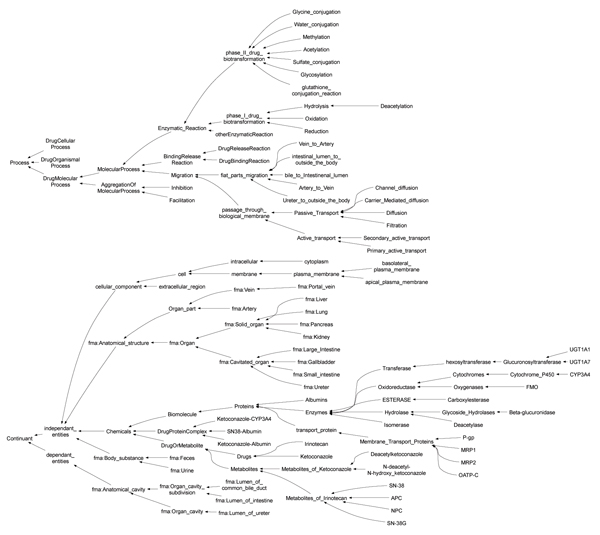
**Class hierarchy for controlled vocabularies of DIO**. Classes prefixed with "fma:" were references for the classes defined in the FMA. The class hierarchy was stored in "dio_event.owl". According to BFO, the controlled vocabulary was divided into two parts; process (e.g. events) and continuants (e.g. things). (This figure was generated using ontoviz plug-in of Protege with manual changes.).

One of the unique features of our knowledge-base design, i.e. DIO, is the adoption of the triplet view: intension, extension, attribute for molecular events and pathways as seen in Figure 2. Intension defines the kinds of molecular events and pathways as controlled vocabulary of processes. Attribute defines the components of molecular events and pathways as controlled vocabulary of continuants (see Figure 3 for a part of class hierarchy of controlled vocabulary for drug interaction). These controlled vocabularies are constructed from the viewpoints of "process" and "continuant" as proposed in Basic Formal Ontology (BFO) [9,10]. Molecular event objects are asserted in extension using the controlled vocabularies defined in intention and attributes. As for extension, prototype object modeling is adopted to represent pathway as aggregation of molecular events. This is because an infinite number of terms or classes are required to express all combinations of molecular events. We avoid this problem by treating pathways as dynamic objects deduced from prototype molecular event objects rather than treating them as instances of pathway classes.

#### Dynamic pathway reconstruction and drug interaction detection

The drug metabolic pathway, due to its dynamic nature, is difficult to define a priori in the manner seen in biomolecular metabolic pathways in the Kyoto Encyclopedia of Genes and Genomics (KEGG) [11]. Therefore, the OHA framework provides the Pathway Object Constructor (POC) and Drug Interaction Detector (DID). Pathway Object Constructor dynamically deduces pathways from the DIO, depending on contexts or situations. Drug Interaction Detector detects drug interaction candidates finding intersections from those generated pathways.

These inferences are mapped onto the Drug Interaction Ontology as hypothetical assertions. The generated pathways and detected drug interactions are asserted as aggregation of molecular events. The detected drug interactions can be segmentalized such as competitive inhibition, noncompetitive inhibition, and uncompetitive inhibition when binding site information is available. This is effective to select an appropriate differential equation depending on the inhibition model.

#### Numerical simulation

In order to incorporate a quantitative simulation system into the OHA framework, the following two aspects were considered: a methodology for automatic conversion from a generated pathway to a quantitative simulation model, and a methodology for the validation of numerical simulation models by analysing the dependences of kinetic parameters and physiological parameters. By solving these two issues, the OHA framework can can be applied to *in silico *prediction of individual drug interactions for multiple-drug regimens, assuming that kinetic parameters and the initial enzyme concentration are roughly estimated by individual genetic variations and health indices of bio-markers.

A simulation model is automatically generated from a pathway generated by the Pathway Object Constructor and Drug Interaction Detector. The pathway is converted to intermediate model by merging organs and molecular events, respectively, to fit a given simulation model framework such as compartment model. Differential equations for the simulation model are generated from the intermediate model by converting the merged events to mathematical expression.

We used the parameter-parameter dependency analysis system (PPD Viewer) designed by Konagaya et al. [12], with high-throughput numerical simulation engine and interactive visualization tools developed on OBIGrid [3,13-16]. The system can predict the concentration/time profiles and those moment parameters such as area under the curve (AUC), area under the moment curve (AUMC), and mean resident time (MRT) when changing some kinetic parameters in the range of one thousandth to thousands of physiological conditions. The system visualizes dependencies among kinetic parameters and molecular concentrations in terms of moment parameters of a compound in target organs.

The generated simulation models and pharmacokinetic moment parameter values are mapped onto the Drug Interaction Ontology as hypothetical assertions. The simulation models are asserted as aggregations of objects representing terms and parameters in differential equations. Those objects have references to the components of the pathway objects from which the simulation models were generated. The moment parameter values are asserted with the drug interaction objects and the corresponding simulation model for the further analysis.

### Interaction between Irinotecan and Ketoconazole

Irinotecan (CPT-11) is an anti cancer drug which is commonly used for colon and breast cancers [17]. Irinotecan is a prodrug of SN-38, anti neoplastic topoisomerase I inhibitor, and is bioactivatied by carboxyl esterase (CE) [17]. About 60% of irinotecan is excreated as unchanged drug from bile and kidney [18]. Irinotecan is also metabolised by CYP3A4 to form APC and NPC [17]. NPC is further metabolized by CE to form SN-38. SN-38 undergoes glucronidation by UGT1A1 to form the inactive glucronide, SN-38G [17]. In addition, it is known that the mutations on UGT1A1, UGT1A1*28 which decreases the expression of UGT1A1 enzyme down to 30%, has strong relationship with some side effects of irinotecan [19,20]. Ketoconazole (KCZ) is an anti fungal drug and a well known inhibitor of CYP3A4. Ketoconazole undergoes extensive metabolism in the liver to form several metabolites [21,22]. About 2 to 4% of urinary radioactivity represents unchanged drug [22].

It has been reported that the inhibition of CYP3A4 by ketoconazole influences the metabolism of irinotecan, which results in 6% SN-38 increase [23].

## Results and discussion

### Results

#### Pathway generation and drug interaction detection

**Figure 4:**
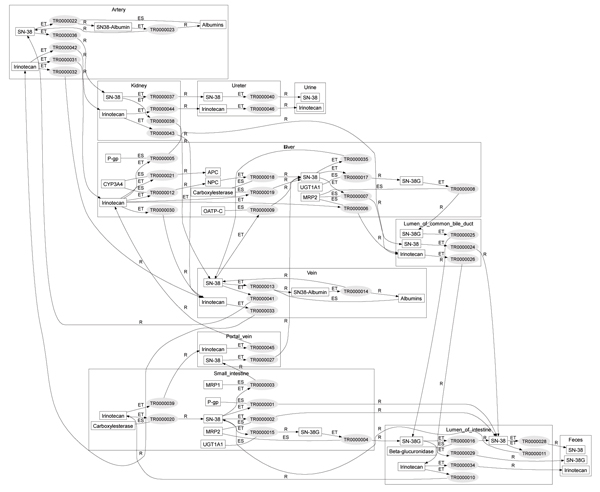
**Inferred pathway of irinotecan**. Small rectangles and ellipses are instances of continuants and molecular events, respectively. Large rectangles such as small intestine are the compartments. Molecular event and trigger, situator, and resultant were connected by solid lines. Each molecular event was connected by the shared continuants. For example, TR0000020 and TR0000002 in the small intestine were connected because they share the same instance of SN-38. This connection can be interpreted as that irinotecan is metabolized into SN-38 (TR0000020) and then SN-38 is transported to the intestinal lumen. (This figure was generated by our inference system.).

The pathways of intravenously administered irinotecan and orally administered ketoconazole were inferred as aggregation of molecular events by the Pathway Object Constructor. Figure 4 shows the generated irinotecan metabolic pathway object where irinotecan and its derivatives circulate through the veins, liver, bile, intestines, and portal vein, namely, the enterohepatic circulation, and are excreted through the kidneys or through the bile. These generated pathways are consistent with in vivo studies [17]. The generated pathway object is sound in the sense that the object is deduced from the Drug Interaction Ontology (DIO) represented by Web Ontology Language (OWL-DL) [24].

**Figure 5:**
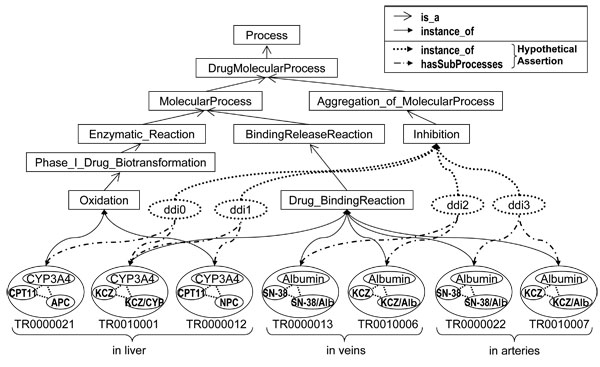
**Dynamically asserted instances of interactions**. Rectangles are owl classes corresponding to intension. Circles are owl instances corresponding to molecular events and attribute. Detected drug interactions were hypothetically asserted as owl instances (dotted circles) along with hasSubprocess properties (dotted and dashed edges).

Interactions between intravenously administered irinotecan and orally administered ketoconazole were detected and asserted by the Drug Interaction Detector. The detected drug interactions and the hypothetic assertion are shown in Figure 5. The assertion contains four drug interactions; two of them concern "drug binding reaction" to albumin in veins (ddi2) and arteries (ddi3), and the rest of them concern "oxidation" by CYP3A4 (ddi0 and ddi1). The detected drug interaction concerning CYP3A4 (ddi0 and ddi1) has been confirmed by the literature on in vivo studies [23].

#### Numerical simulation

**Figure 6:**
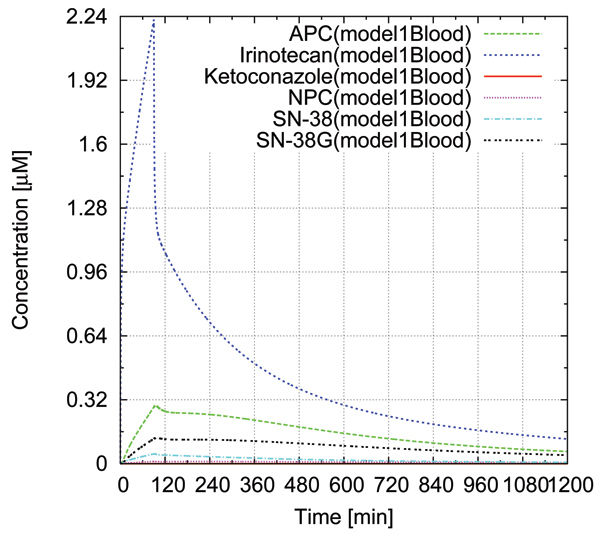
**Blood Concentration/time profile of irinotecan, SN-38, APC, NPC, and SN-38 in blood compartment for solo administration of irinotecan**. The drug concentrations in blood without ketoconazole administration was estimated by numerical simulation. Intravenous drip infusion (125 *mg*/*m*^2^, 90 min) was assumed for irinotecan administration.

We evaluated the effects of drug interactions concerning CYP3A4 quantitatively with numerical simulations. Three simulations were performed: sole administration of irinotecan for a patient having UGT1A1*1/*1 (wild type), co-administration of irinotecan and ketoconazole for a patient having UGT1A1*1/*1, and sole administration of irinotecan for a patient with UGT1A1*28/*28 which decreases the expression of UGT1A1 down to 30% than UGT1A1*1/*1. Intravenous drip infusion (125 *mg*/*m*^2^, 90 min) was assumed for irinotecan, and oral administration (200 mg) was assumed for ketoconazole. Figure 6 shows the simulated concentration/time profiles of irinotecan, SN-38, APC, NPC, SN-38G in blood for the simulation of sole administration of irinotecan for a patient having UGT1A1*1/*1. The concentration/time profiles agree with experimental data reported by Slatter et al. [18].

Table 1 shows the comparisons of AUC and Cmax for the simulations. By the ketoconazole administration, the AUC of APC and NPC were decreased to 48.1% and 35.3%, respectively. The AUC of SN-38 were increased only to 108% by the ketoconazole administration. Similarly, the Cmax of APC and NPC were decreased to 25.6% and 20.2%, respectively, whereas the Cmax of SN-38 was increased to 105% by the ketoconazole administration. On the other hand, in case of the UGT1A1*28/*28 mutation, the AUC and Cmax of SN-38 were significantly increased: the AUC was increased to 208% and the Cmax was increased to 165%. This implies that patients with UGT1A1*28/*28 may suffer severe side effects when the doses are same as patients with UGT1A1*1/*1. These results agree with the previously published experimental papers by Kehrer et al. [23], Sai et al. [19], and Ando et al. [20]. In addition, the simulation results were asserted into the ontology along with the simulation model.

**Table 1:** AUC and Cmax of blood concentration/time profile obtained by numerical simulations.

	Irinotecan	APC	NPC	SN-38	SN-38G
*AUC*_*KCZ*- _[nmol·min/ml]	672	233	14.2	29.8	137
*Cmax*_*KCZ*- _[*μ*M]	2.22	0.290	0.0113	0.0491	0.129
*AUC*_*KCZ*+ _[nmol·min/ml]	721	112	5.01	32.2	147
*Cmax*_*KCZ*+ _[*μ*M]	2.29	0.0741	0.00228	0.0515	0.136
*AUC*_*KCZ*+_/*AUC*_*KCZ*- _[%]	107	48.1	35.3	108	107
*Cmax*_*KCZ*+_/*Cmax*_*KCZ*- _[%]	103	25.6	20.2	105	105

*AUC*_*UGT *_[nmol·min/ml]	672	233	14.2	62.1	85.5
*Cmax*_*UGT *_[*μ*M]	2.22	0.290	0.0113	0.0812	0.0693
*AUC*_*UGT*_/*AUC*_*KCZ*- _[%]	100	100	100	208	62.4
*Cmax*_*UGT*_/*Cmax*_*KCZ*- _[%]	100	100	100	165	53.7

AUC and Cmax values for solo administration of irinotecan (*AUC*_*KCZ*-_, *Cmax*_*KCZ*-_), co-administration of irinotecan and ketoconazole (*AUC*_*KCZ*+_, *Cmax*_*KCZ*+_), and sole administration of irinotecan with patients having UGT1A1*28/*28 (*AUC*_*UGT*_, *Cmax*_*UGT*_) were obtained.

### Discussion

#### Pathway reconstruction and drug interaction detection

The OHA framework automatically generated pathways for co-administration of irinotecan and ketoconazole, and detected drug interactions. The generated pathways and two of the drug interactions involving CYP3A4 were confirmed by published papers. The detected interactions with plasma albumin (ddi2 and ddi3) have not been reported in any literature as far as we know. One possible reason is that the effects of drug interactions may be negligible because of the high plasma albumin concentration compared to drug concentration. To distinguish serious drug interactions from those negligible ones, a qualitative evaluation, such as numerical simulation, is necessary.

#### Numerical simulation

The simulation model succeeded to reproduce Slatter's experimental data of irinotecan dosage to a large extent except for the Cmax of irinotecan blood concentration, cyclic fluctuation of irinotecan concentration and a bile cancer patient data.

The Cmax of drug concentrations in the simulation model strongly depend on renal clearance, bile clearance, and kinetic parameters of enzymes. Because these parameters were estimated from the recovery amount data in urine and feces by Slatter et al. [18] and *in vitro *experiments data reported as published papers [25-29], the excess of irinotecan's Cmax in the simulation model might be explained by the false peak in the experimental data which results from time lag of sampling

Another possibility is the effect of reabsorption in intestine. The time course of the experimental data indicate slight increase of SN-38G blood concentration in the period of 12 hours. This may result from the reabsorption of SN-38 thorough small intestine. In order to include the reabsorption process into the simulation, the kinetic parameters in bacterial flora in the intestine and amount/time profile data of bile excretion are required.

Lastlym Slatter et al. reported that one patient with a bile cancer showed different bile and urine recovery pattern from other patients [18]. Virtual population [30] may be helpful to reconstruct this behaviour.

## Conclusion

Deduction of hypothetic drug interactions from the Drug Interaction Ontology was demonstrated for an irinotecan plus ketoconazole regimen. The prototype system detected four drug interactions. Two of them concerned cytochrome p450 (CYP3A4) and were consistent with known drug interactions. The other two concerned albumin, the effects of the interactions would be negligible as long as the drug concentration is low. We then quantitatively examined the effect of the drug interactions concerned CYP3A4 and the effect of genetic variation UGT1A1*28 using numerical simulation. The numerical simulation indicates that the drug interaction has only a limited effect on the pharmacokinetics of SN-38 for an irinotecan plus ketoconazole regimen. However, the genetic variation on UGT1A1 showed two-fold increase of SN-38's AUC.

Finally, in order to realize *in silico *prediction of drug interactions, the following future works are remained: expansion of the Drug Interaction Ontology for other drugs, incorporation of virtual population model for generic variation analysis, and refinements of the pathway generation rules, drug interaction detection rules, and the numerical simulation models.

## Methods

### The OHA framework

**Figure 7:**
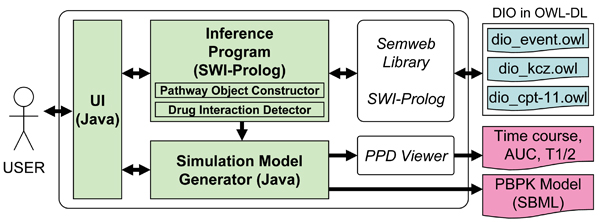
**Overview of the prototype system**. User interface (UI), inference program, and visualization program were implemented in Prolog and JAVA. The ontology (DIO) was divided into three OWL files. 1) Class hierarchy and commonly used instances are stored in dio_event.owl. 2) Instances about irinotecan and its pathway are stored in dio_cpt-11.owl. 3) Instances about ketoconazole and its pathway are stored in dio_KCZ.owl.

We implemented the following prototype system to prove the concept of the OHA framework. The system was designed to predict potential drug interactions occurring after concomitant administration of irinotecan and ketoconazole by comparing the drug metabolic pathways generated from primitive molecular events. Figure 7 shows an overview of the prototype system. The system consists of the knowledge base for DIO, the inference programs of the Pathway Object Constructor and Drug Interaction Detector, simulation model generator, and simulation engine.

DIO, shown in Figure 2, was written in OWL-DL; the controlled vocabularies of process and continuant were implemented as OWL class hierarchy, and extension and part of attributes, including molecules and organs, were implemented as OWL instances. The molecular event objects in extension were represented by OWL instances and OWL properties. The ontology referred to other taxonomies and ontologies for well-established vocabularies of biochemical terms, anatomical entities and properties. This enabled the reduction of our ontology construction cost and to concentrate our efforts on the information specific to drug interaction.

The ontology for irinotecan and ketoconazole was built with Protege [31]. The information about these drugs has been collected from various articles [17,22,32-38]. The total number of classes and instances of the current DIO is 178 and 143, respectively. Approximately two thirds of the classes were mapped to the Unified Medical Language System (UMLS) [39] and/or Foundational Model of Anatomy (FMA) [40,41]; 106 classes were mapped to the UMLS; 32 classes were mapped to the FMA. The class hierarchy is shown in Figure 3. Twenty-six properties were imported from the Open Biomedical Ontology Relation Ontology (OBO Relation) [42]; and five properties were newly defined to implement molecular event objects as OWL instances (Table 2). The ontology OWL files: dio_event.owl, dio_cpt-11, and dio_KCZ.owl, are available as additional files 1, 2, 3.

**Table 2:** The major properties of molecular event objects in OWL-DL.

Properties	Domain (class)	Range (class)
oboRel:relationship	Process, Continuant	Process, Continuant
-oboRel:located in	independent_entities	Independent_entities, fma:Anatomical_cavity
-oboRel:has_participant	Process	Independent_entities
-- hasResultantParticipant	Process	Independent_entities
-- hasTriggerParticipant	Process	Independent_entities
-- hasSituatedParticipant	Process	Independent_entities

hasSubProcesses	Process	Process
occurred_in	Process	Independent_entities, fma:Anatomical_cavity

The relations among molecular events, attributes (continuants) and drug interactions shown in Figure 2 were implemented as OWL properties. We defined five properties for implementing molecular event objects in OWL-DL in addition to the properties of OBO Relation ontology. Three properties for resultant, trigger, and situated participants were defined as sub properties of oboRel:has_participant, inheriting the definition of the super property. Note that classes for Domain and Range of properties are denoted by the controlled vocabulary defined in Figure 3.

The inference programs of the Pathway Object Constructor and Drug Interaction Detector were implemented with SWI-Prolog [43] and the Semweb library. The Semweb library enables the system to access OWL and RDF files as Prolog clauses. All logical inferences, including pathway generation and drug interaction detection, were implemented in Prolog.

Systems for simulation model generation, and pathway visualization were implemented in JAVA. These systems access DIO through SWI-Prolog semweb library to get detailed information of the generated pathways. The simulation models were generated in the form of SBML, then translated in the form of Octave. Graphviz [44], a graph visualization program designed by Gansner et al., was used to visualize pathways generated by the inference programs. The PPD Viewer designed by Azuma et al. [12] was used for numerical simulation.

### Numerical simulation model for co-administration of Irinotecan and Ketoconazole

In order to increase predictive performance, a simplified pathway was used for the generation of simulation models from the viewpoint of the trade off between model complexity and data availability. Reabsorption through small intestine and reactions concerning albumin were omitted due to the lack of information. Furthermore, enzymatic reactions involved metabolism of ketoconazole were integrated into a single enzymatic reaction based on the work of Chien et al. [29].

A simulation model was automatically generated from the simplified pathway. The organs and tissues were integrated into 8 compartments, i.e. blood (including rapidly equilibrating tissues: artery, heart, kidneys, lung, and veins), liver, GI (gastrointestinal consists of large intestine, small intestine, portal vein, and stomach), adipose, NET (non-eliminating tissue such as skin and muscle), GI lumen, bile lumen, and urine. Michaelis-Menten equations were used for all enzymatic reactions. We used a competitive Michaelis-Menten inhibition model for this simulation as used for midazolam and ketoconazole inhibition by Chien et al. [29]. For drugs and their metabolites in blood, adipose, NET, GI, and liver, the following equations apply:

(1) $$\begin{array}{c} \frac{dC_{1D}}{dt}=\{-CL_{u,r,D}f_{BD}C_{1D}+(Q_{2}+Q_{3})\frac{C_{2D}}{Kp_{2D}}+Q_{5}\frac{C_{5D}}{Kp_{5D}}\\ +Q_{4}\frac{C_{4D}}{Kp_{4D}}-(Q_{2}+Q_{3}+Q_{4}+Q_{5})C_{1D}\}/V_{1} \end{array}$$

(2) $$\frac{dC_{4D}}{dt}=\left(Q_{4}C_{1D}-Q_{4}\frac{C_{4D}}{Kp_{4D}}\right)/V_{4}$$

(3) $$\frac{dC_{5D}}{dt}=\left(Q_{5}C_{1D}-Q_{5}\frac{C_{5D}}{Kp_{5D}}\right)/V_{5}$$

(4) $$\frac{dC_{3IM}}{dt}=\left(Q_{3}C_{1IM}-Q_{3}\frac{C_{3IM}}{Kp_{3IM}}\right)/V_{3}$$

(5) $$\frac{dC_{3KCZ}}{dt}=\left(ka_{KCZ}X_{KCZ,GILumen}+Q_{3}C_{1KCZ}-Q_{3}\frac{C_{3KCZ}}{Kp_{3KCZ}}\right)/V_{3}$$

(6) $$\begin{array}{c} \frac{dC_{2I}}{dt}=\{Q_{2}C_{1I}+Q_{3}\frac{C_{3I}}{kp_{3I}}-(Q_{2}+Q_{3})\frac{C_{2I}}{kp_{2I}}\\ -(CL_{u,b,I}+\frac{Vmax_{a}alpha_{a}V_{2}}{km_{a}+\frac{fb_{I}C_{2I}}{Kp_{2I}}}+\frac{Vmax_{c}alpha_{c}V_{2}}{km_{c}(1+\frac{fb_{KCZ}C_{2KCZ}}{Kp_{2KCZ}Ki_{APC}})+\frac{fb_{I}C_{2I}}{Kp_{2I}}}\\ +\frac{Vmax_{d}alpha_{d}V_{2}}{km_{d}(1+\frac{fb_{KCZ}C_{2KCZ}}{Kp_{2KCZ}Ki_{NPC}})+\frac{fb_{I}C_{2I}}{Kp_{2I}}})\frac{fb_{I}C_{2I}}{Kp_{2I}}\}/V_{2} \end{array}$$

(7) $$\begin{array}{c} \frac{dC_{2APC}}{dt}=\{Q_{2}C_{1APC}+Q_{3}\frac{C_{3APC}}{Kp_{3APC}}+\frac{Vmax_{c}alpha_{c}V_{2}}{Km_{c}(1+\frac{fb_{KCZ}C_{2KCZ}}{Kp_{2KCZ}Ki_{APC}})+\frac{fb_{I}C_{2I}}{Kp_{2I}}}\frac{fb_{I}C_{2I}}{Kp_{2I}}\\ -(Q_{2}+Q_{3})\frac{C_{2APC}}{Kp_{2APC}}-CL_{u,b,APC}\frac{fb_{APC}C_{2APC}}{Kp_{2APC}}\}/V_{2} \end{array}$$

(8) $$\begin{array}{c} \frac{dC_{2NPC}}{dt}=\{Q_{2}C_{1NPC}+Q_{3}\frac{C_{3NPC}}{Kp_{3NPC}}+\frac{Vmax_{d}alpha_{d}V_{2}}{Km_{d}(1+\frac{fb_{KCZ}C_{2KCZ}}{Kp_{2KCZ}Ki_{NPC}})+\frac{fb_{I}C_{2I}}{Kp_{2I}}}\frac{fb_{I}C_{2I}}{Kp_{2I}}\\ -(Q_{2}+Q_{3})\frac{C_{2NPC}}{Kp_{2NAPC}}-\left(CL_{u,b,NPC}\frac{Vmax_{b}alpha_{b}V_{2}}{Km_{b}+\frac{fb_{NPC}C_{2NPC}}{Kp_{2NPC}})}\right)\frac{fb_{NPC}C_{2NPC}}{Kp_{2NPC}}\}/V_{2} \end{array}$$

(9) $$\begin{array}{c} \frac{dC_{2SN-38}}{dt}=\{Q_{2}C_{1SN-38}+Q_{3}\frac{C_{3SN-38}}{Kp_{3SN-38}}-(Q_{2}+Q_{3})\frac{C_{2SN-38}}{Kp_{2SN-38}}\\ +\frac{Vmax_{a}alpha_{a}V_{2}}{Km_{a}+\frac{fb_{I}C_{2I}}{Kp_{2I}}}\frac{fb_{I}C_{2I}}{Kp_{2I}}+\frac{Vmax_{b}alpha_{b}V_{2}}{Km_{b}+\frac{fb_{NPC}C_{2NPC}}{Kp_{2NPC}}}\frac{fb_{NPC}C_{2NPC}}{Kp_{2NPC}}\\ -\left(CL_{u,b,SN-38}+\frac{Vmax_{f}alpha_{f}V_{2}}{Km_{f}+\frac{fb_{SN-38}C_{2SN-38}}{Kp_{2SN-38}}}\right)\frac{fb_{SN-38}C_{2SN-38}}{Kp_{2SN-38}}\}/V_{2} \end{array}$$

(10) $$\begin{array}{c} \frac{dC_{2SN-38G}}{dt}=\{Q_{2}C_{1SN-38G}+Q_{3}\frac{C_{3SN-38}}{Kp_{3SN-38}}+\frac{Vmax_{f}alpha_{f}V_{2}}{Km_{f}+\frac{fb_{SN-38}C_{2SN-38}}{Kp_{2SN-38}}}\frac{fb_{SN-38}C_{2SN-38}}{Kp_{2SN-38}}\\ -(Q_{2}+Q_{3})\frac{C_{2SN-38G}}{Kp_{2SN-38G}}-CL_{u,b,SN-38G}\frac{fb_{SN-38G}C_{2SN-38G}}{Kp_{2SN-38G}}\}/V_{2} \end{array}$$

(11) $$\begin{array}{c} \frac{dC_{2KCZ}}{dt}=\{Q_{2}C_{1KCZ}+Q_{3}\frac{C_{3KCZ}}{Kp_{3KCZ}}-(Q_{2}+Q_{3})\frac{C_{2KCZ}}{Kp_{2KCZ}}\\ -\frac{Vmax_{e}alpha_{e}V_{2}}{Km_{e}+\frac{fb_{KCZ}C_{2KCZ}}{Kp_{2KCZ}}}\frac{fb_{KCZ}C_{2KCZ}}{Kp_{2KCZ}}\}/V_{2} \end{array}$$

Here *Qp*_*n*_, and *V*_*n *_(*n *= 1 to 5) are blood flows and volume of compartments, respectively. The subscripts *n *stands for compartments: *n *= 1, 2, 3, 4 and 5 represents blood, liver, GI, adipose, and NET, respectively. *Kp*_*ns*_, and *C*_*ns *_(*n *= 1 to 5, *s *= D, I, KCZ, IM, APC, NPC, SN-38, and SN-38G) are tissue-to-blood concentration ratios, and blood concentrations, respectively. The subscripts *s *stands for drugs and their metabolites: *s *= D, I, KCZ, IM, APC, NPC, SN-38, and SN-38G represents drugs and metabolites, irinotecan, ketoconazole, metabolites of irinotecan, APC, NPC, SN-38, and SN-38, respectively. *CL*_*u*,*r*,*s *_*CL*_*u*,*b*,*s*_, and *f*_*Bs *_(*s *= D, I, KCZ, IM, APC, NPC, SN-38, and SN-38G) are renal clearance for unbound drugs, bile clearance for unbound drugs, and blood unbound fraction, respectively. *ka*_*KCZ *_and *X*_*KCZ*;*GILumen *_are absorption rate constant of ketoconazole and amount of ketoconazole in the GI luminal compartment, where ketoconazole is absorbed. *V max*_*m*_, *km*_*m*_, and *α*_*m *_(*m *= a, b, c, d, e, and f) are Vmax, Michaelis constant, and expression amount of enzyme, respectively. The subscripts *m *stands for enzymatic reactions: m = a, b, c, d, e, and f represents metabolism of irinotecan to form SN-38 by CE, metabolism of NPC to form SN-38 by CE, oxidation of irinotecan to form APC by CYP3A4, oxidation of irinotecan to form NPC by CYP3A4, glucronidation of SN-38 to form SN-38G by UGT1A1, and metabolism of ketoconazole by CYP3A4, respectively. *Ki*_*APC *_and *Ki*_*NPC *_are inhibition constants. During the drip infusion of irinotecan, the following equation apply for blood concentration of irinotecan.

(12) $$\begin{array}{c} \frac{dC_{1I}}{dt}=\{k0_{I}-CL_{u,r,I}f_{BI}C_{1I}+(Q_{2}+Q_{3})\frac{C_{2I}}{Kp_{2I}}+Q_{5}\frac{C_{5I}}{Kp_{5I}}\\ +Q_{4}\frac{C_{4I}}{Kp_{4I}}-(Q_{2}+Q_{3}+Q_{4}+Q_{5})C_{1I}\}/V_{1} \end{array}$$

Here *k*0_*I *_is a constant for the velocity of drip infusion.

The values of physiologic parameters, kinetic parameters, and inhibition constants and administration parameters for the simulation model are shown in Table 3, 4, 5 and 6 respectively. The values of these parameters were based on published papers except enzyme expression parameters (*α*) and tissue-to-blood concentration ratios (Kp). The values for *α *and Kp were obtained by fitting the simulation result to the experimental data published by Slatter et al. [18].

**Table 3:** Kinetic parameters for the protein binding, tissue distribution, and urinary and biliary excretion of ketoconazole, irinotecan, and their metabolites.

Compound	*f*_*B*_	*Kp*_*GI*_	*Kp*_*Liver*_	*Kp*_*Adipose*_	*Kp*_*NET*_	*CL*_*U*,*R *_[mL/min/kg]	*CL*_*U*,*B *_[mL/min/kg]
Irinotecan	0.37	1.00	1.00	10.0	3.00	6.15	10.6
APC	0.37	1.00	1.00	1.50	0.06	1.47	5.45
NPC	0.37	1.00	1.00	6.00	2.00	1.49	14.5
SN-38	0.05	1.00	1.00	2.00	0.70	9.91	103
SN-38G	1.00	1.00	1.00	2.80	0.08	1.44	2.03

KCZ	0.01	1.00	1.00	15.0	1.50	130	-

Blood unbound fraction (*f*_*B*_) values for irinotecan and SN-38 were obtained from the published paper by Oliver et al. [45]. The *f*_*B *_values for NPC and APC were assumed to be the same as that of Irinotecan. *f*_*B *_value for SN-38G was assumed to be 1.00. Urinary and biliary clearances (*CL*_*U*,*R*_, *CL*_*U*,*B*_) were defined for unbound blood concentration. *CL*_*U*,*R *_and *CL*_*U*,*B *_values for irinotecan, APC, SN-38, SN-38G were calculated using data from the publish paper [18]. Kp values were determined so that the simulation concentration/time profile fits to the experimental data from Slatter et al. [18].

**Table 4:** Kinetic parameters for metabolic enzymes.

Enzyme	Substrate	Product	*K*_*m *_[*μ*M]	*V*_*max *_[pmol/min/mg protein]	*α*[mg protein/g tissue]	*V*_*max*_·*α/K*_*m *_[mL/min/g tissue]
Carboxylesterase	Irinotecan	SN-38	2.30	2.11	128	0.117
Carboxylesterase	NPC	SN-38	2.30	2.11	128	0.117
CYP3A4	Irinotecan	APC	18.4	26.0	73.3	0.104
CYP3A4	Irinotecan	NPC	48.2	74.1	11.7	0.0180
UGT1A1	SN-38	SN-38G	3.80	50.8	750	10.0

CYP3A4	KCZ	MOK	0.00810	12.5	44.3	68.4

Because of the lack of experimental data, *K*_*m *_and *V*_*max *_values for metabolism of NPC to SN-38 by CE were assumed to be the same as those for metabolism of irinotecan to SN-38 by CE. *K*_*m *_and *V*_*max *_values for other reactions were obtained from the published papers [25–29]. Values of unknown parameter *α *were determined so that the simulation concentration/time profile fit to the experimental data published by Slatter et al. [18]. The relatively high fitted value for *α *in the glucronidation of SN-38 may result from the increase in UGT1A1 expression as drug response. The relatively low value for *α *in the oxidation of irinotecan to NPC by CYP3A4 suggests a competition in this reaction with the oxidation of irinotecan to APC by CYP3A4.

**Table 5:** Physiologic parameters.

Organ	Blood flow rate [mL/min/kg]	Volume [mL/kg]
Blood	61.1	51.0
Liver	5.79	32.3
GI	13.4	32.1
Adipose	4.45	204
NET	37.4	681

The physiologic parameters were obtained from the work by Willmann et al. [30]. These values assumes Caucasian male with body weight of 73 kg and height of 176 cm.

## Competing interests

The authors have received no financial support or other benefits from commercial sources for the work reported in the manuscript, and no other financial interests that any of the authors may have could create a potential conflict of interest or the appearance of a conflict of interest with regard to the work.

## Authors' contributions

Takeshi Arikuma carried out the machine inference studies, participated in Drug Interaction Ontology development, participated in numerical simulation studies and drafted the manuscript. Sumi Yoshikawa carried out the ontology studies, participated in machine inference studies, and helped to draft the manuscript. Ryuzo Azuma carried out the numerical simulation and visualization studies. Kazumi Matsumura extracted knowledge from the articles for the Drug Interaction Ontology when she worked for RIKEN GSC. Kentaro Watanabe developed the original drug-interaction prediction system when he studied in TITECH. Akihiko Konagaya conceived of the study, partcipated in its design and coordination, and helped to draft the manuscript.

**Table 6:** Parameters for drug interactions and drug administrations.

Parameters	Values
*Ki*_*APC*_	1.40 * 10^-3^
*Ki*_*NPC*_	5.94 * 10^-4^
*ka*_*KCZ *_[*min*^-1^]	1.83 * 10^-2^
*dose*_*Irinotecan *_[nmol/kg]	4.86 * 10^3^
*t*_*dose*,*Irinotecan *_[min]	90
*dose*_*KCZ *_[nmol/kg]	5.16 * 10^3^

*Ki*_*APC *_and *Ki*_*NPC *_were calculated from the experimental data reported by Haaz et al. [25,26]. *ka*_*KCZ *_was obtained the from published paper [29].

## Supplementary Material

Additional file 1 owl file for dio_event ontology: This file includes all OWL classes and properties defined for the drug interaction ontology (DIO). Click here for file

Additional file 2 owl file for dio_cpt-11 ontology: This file includes all OWL instances of reactions defined for irinotecan. Click here for file

Additional file 3 owl file for dio_kcz ontology: This file includes all OWL instances of reactions defined for ketoconazole. Click here for file
